# Supplementary material for: Limited intestinal inflammation despite diarrhea, fecal viral RNA and SARS-CoV-2-specific IgA in patients with acute COVID-19
Source: medRxiv. 2020 Dec 9:2020.09.03.20183947. Originally published 2020 Sep 5. Preprint. [Version 2] doi: 10.1101/2020.09.03.20183947 (PMC7480054; doi:10.1101/2020.09.03.20183947)
Supplement: Supplement 2020 [file 95609-2020.09.03.20183947-1.pdf]

Britton, Chen-Liaw, Cossarini, Livanos, Spindler *et.al.*

Supplementary Information

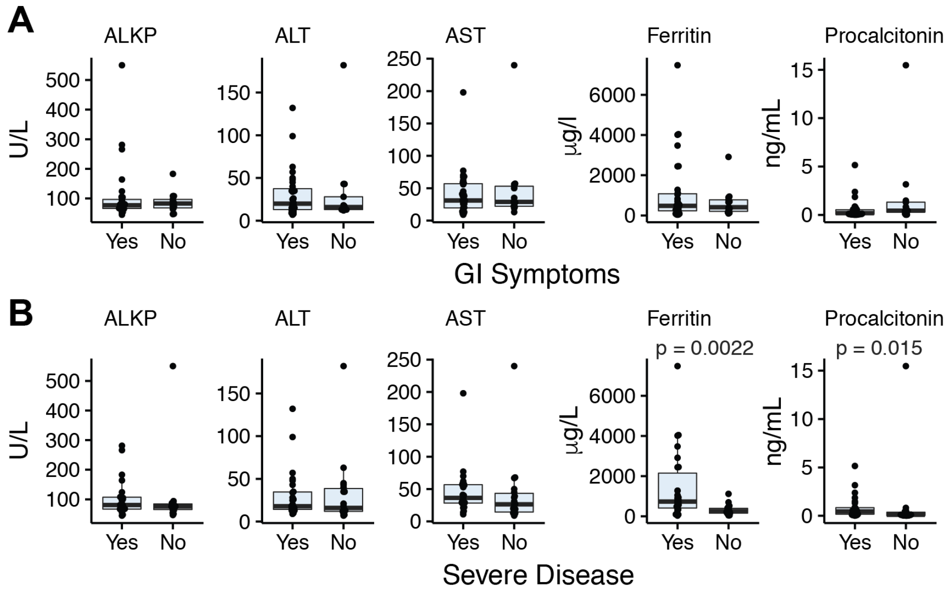

Fig. S1. Gastrointestinal symptoms and serologic parameters in hospitalized COVID-19 patients. **(A)** Liver enzymes and laboratory values in hospitalized COVID-19 patients with and without GI symptoms. **(B)** Liver enzymes and laboratory values in hospitalized COVID-19 patients with severe disease or non-severe disease. Each point represents an individual value for a patient, the box plot shows the median and the interquartile range and the p-values are calculated using the Mann-Whitney test with significance defined as $p<0.05$.

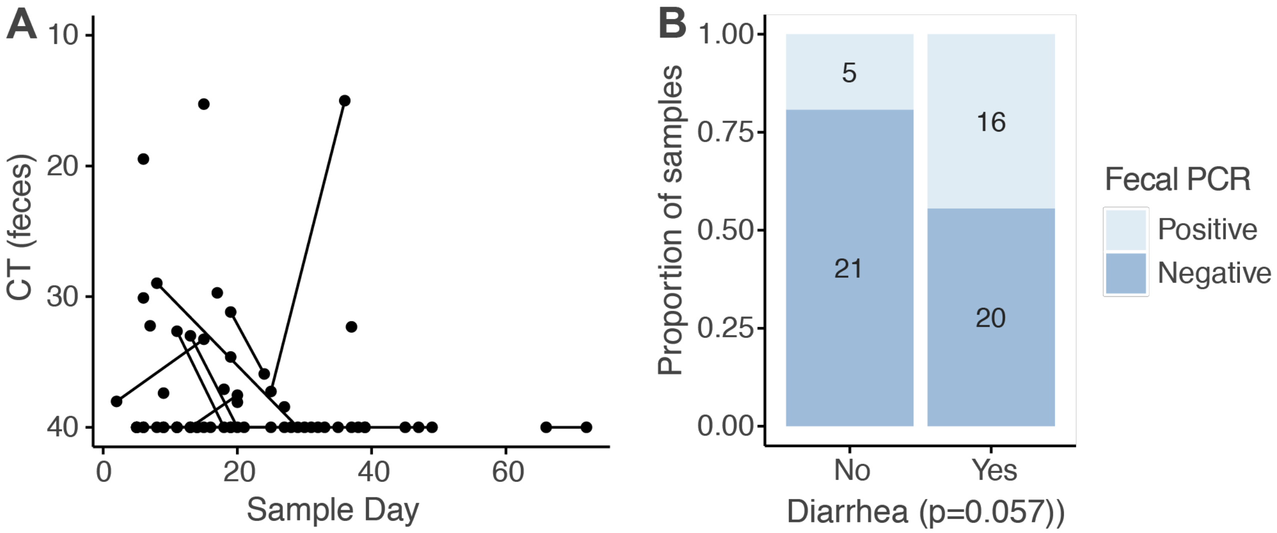

Figure S2. Detection of SARS-CoV-2 virus genome in stool. (A). Each point shows the median SARS-CoV-2 Ct from one sample, plotted against the sampling day relative to the onset of symptoms. Lines connect samples from the same donor. (**B**) The proportion of positive and negative fecal SARS-CoV-2 PCR results from donors with and without diarrhea. p value - Fisher's exact test.

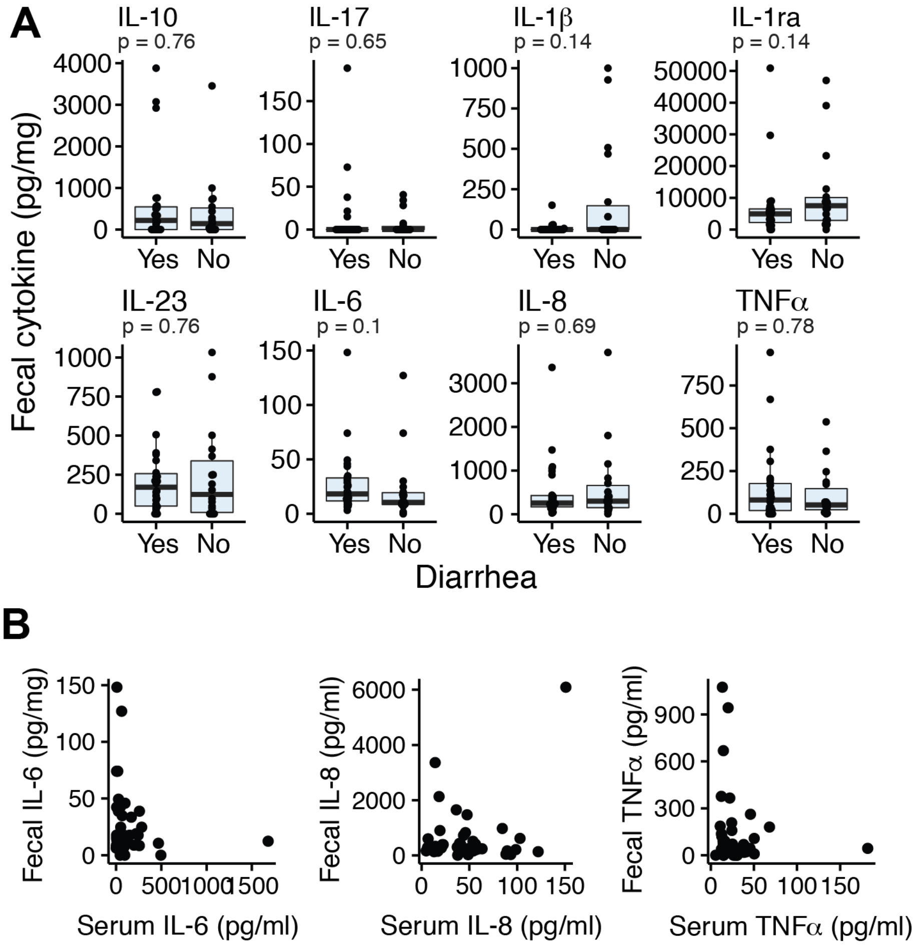

Fig. S3. Fecal cytokine levels in COVID-19 patients. (**A**) Concentrations of the indicated cytokine in fecal samples from COVID-19 patients with and without diarrhea. P values - Mann-Whitney. **(B)** Relationship between concentrations of the indicated cytokine in feces and serum.

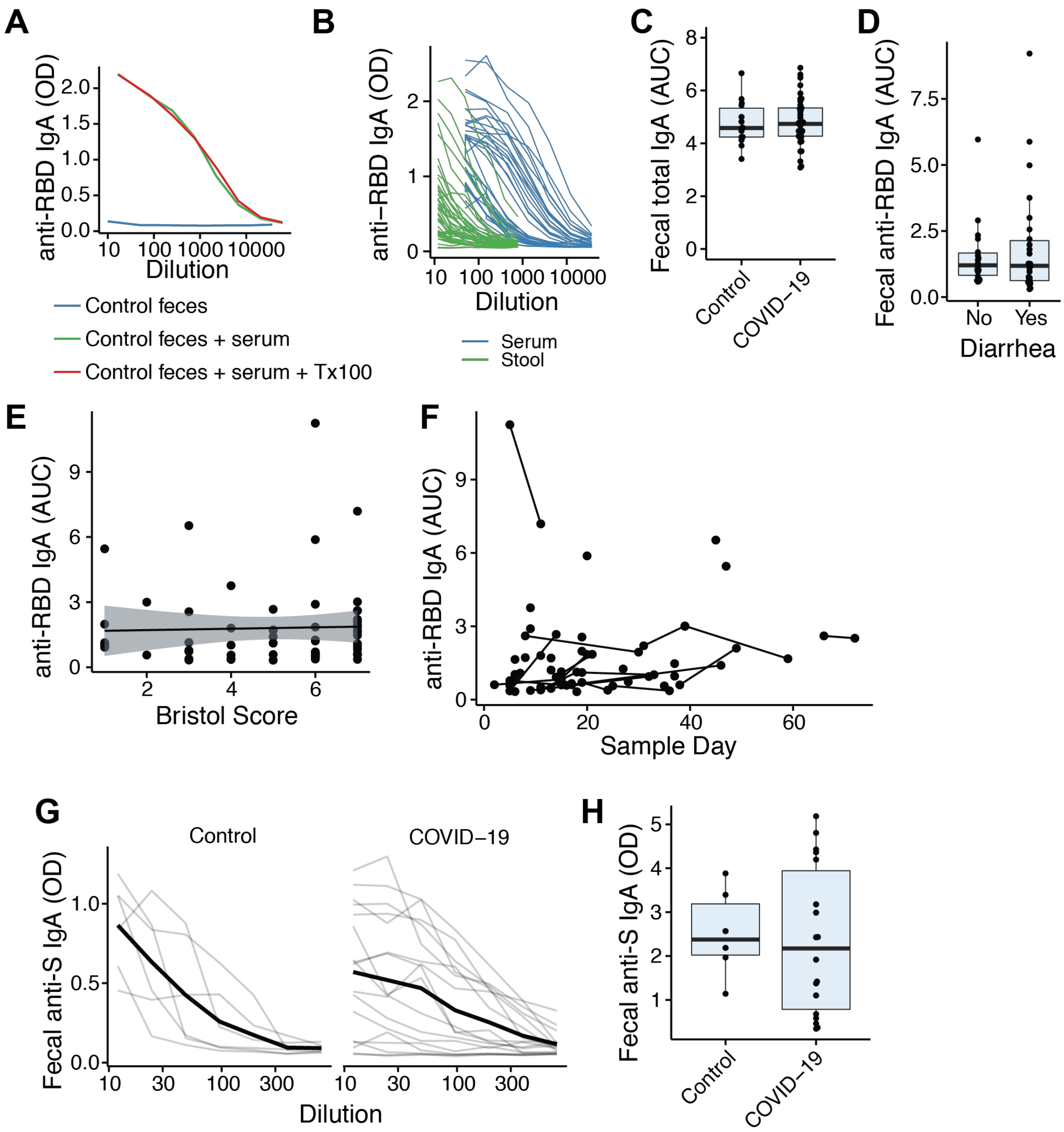

Figure. S4. SARS-CoV-2-specific IgA in stool of COVID-19 patients. (A) Triton X-100 does not reduce detection of anti-RBD IgA in a serum sample from a seropositive donor serum spiked into a healthy donor stool sample. **(B)** Relative titers of serum and fecal anti-RBD IgA in samples from COVID-19 patients. **(C)** Relative titers of total IgA in feces of control donors and COVID-19 patients. (**D** and **E)** Anti-SARS-CoV-2 RBD specific IgA titers are not different in donors with diarrhea. (D – averaged by donor, E - by sample). **(F)** Anti-SARS-CoV-2 RBD IgA in feces over time relative to reported symptom onset. Each point shows data from one sample and lines connect samples from the same donor. (**G**) Anti-SARS-CoV-2 Spike protein IgA in control donors and COVID-19 patients. (**H**) Relative titers of anti-SARS-CoV-2 Spike protein IgA in control donors and COVID-19 patients.

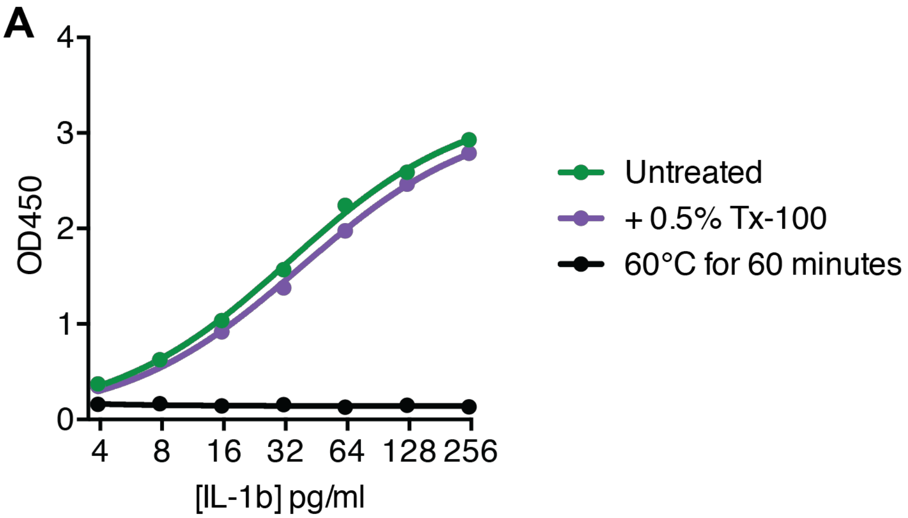

Figure S5. Virus inactivation method compatible with cytokine ELISAs. (A) Recombinant human IL-1β was spiked into a stool homogenate from a healthy donor and either heated at 60°C for 60 minutes or treated with 0.5% Triton X-10 (Tx-100) for 60 minutes at 4°C before ELISA analysis.

Supplementary Table 1: Sample collection and description

The number of each sample type and the day of collection (median +/- the range), relative to the onset of COVID-19 symptoms.

	1st stool sample	2nd stool sample	Serum
Number of samples	44	18	32
Median time in days (range) from symptom onset to sample collection	16 (2-66)	24.5 (11-72)	16 (3-46)

Supplementary Table 2: COVID-19 treatments in patients with and without GI symptoms

The absolute number and percentage of the cohort (in parentheses) who received the indicated therapeutic. Statistical comparisons are by Fisher's exact test.

	Total (n=44)	GI symptoms (n=31)	No GI symptoms (n=13)	p-value
Antibiotics	28 (63.6)	17 (54.8)	11 (84.6)	0.09
Azithromycin	17 (38.6)	8 (25.8)	9 (69.2)	**0.02**
Vancomycin	13 (29.5)	7 (22.6)	6 (46.2)	0.16
Ceftriaxone (CTX)	17 (38.6)	9 (29.0)	8 (61.5)	0.09
Vancomycin + CTX	8 (18.2)	4 (12.9)	4 (30.8)	0.21
Cefepime	12 (27.3)	8 (25.8)	4 (30.8)	0.73
Other antibiotics	10 (22.7)	5 (16.1)	5 (38.5)	0.13
Plasma	20 (45.5)	13 (41.9)	7 (53.8)	0.52
Plasma before any sample collection	19 (43.2)	13 (41.9)	6 (46.2)	>0.99
Hydroxychloroquine	19 (43.2)	12 (38.7)	7 (53.8)	0.51
Remdesivir	13 (29.5)	8 (25.8)	5 (38.5)	0.48
Steroids	18 (40.9)	13 (41.9)	5 (38.5)	>0.99
Therapeutic anticoagulation	31 (70.5)	22 (71.0)	9 (69.2)	>0.99

Supplementary Table 3: Disease severity associated with composite outcome of ICU admission or death

The absolute number and percentage of the cohort (in parentheses) with each indicated COIVD-19 severity. Statistical comparisons are by Fisher's exact test.

**Severity on admission**	ICU admission or death (n=14)	Neither (n=30)	p-value
Mild or moderate	6 (42.9)	22 (73.3)	
Severe	8 (57.1)	8 (26.7)	0.09
**Peak severity**			
Mild or moderate	2 (14.3)	16 (53.3)	
Severe	12 (85.7)	14 (46.7)	**0.02**

Supplementary Table 4: PCR Primers

Primer	Sequence (5'-3')	Reference
2019-nCoV_N1 Forward	GACCCCAAAATCAGCGAAAT	(Lu et al. Emerging Infectious Disease journal 2020)
2019-nCoV_N1 Reverse	TCTGGTTACTGCCAGTTGAATCTG	(Lu et al. Emerging Infectious Disease journal 2020)
2019-nCoV_N1 Probe	ACCCCGCATTACGTTTGGTGGACC	(Lu et al. Emerging Infectious Disease journal 2020)
2019-nCoV_N2 Forward	TTACAAACATTGGCCGCAAA	(Lu et al. Emerging Infectious Disease journal 2020)
2019-nCoV_N2 Reverse	GCGCGACATTCCGAAGAA	(Lu et al. Emerging Infectious Disease journal 2020)
2019-nCoV_N2 Probe	ACAATTTGCCCCCAGCGCTTCAG	(Lu et al. Emerging Infectious Disease journal 2020)
RNase P Forward	AGATTTGGACCTGCGAGCG	(Lu et al. Emerging Infectious Disease journal 2020)
RNase P Reverse	GAGCGGCTGTCTCCACAAGT	(Lu et al. Emerging Infectious Disease journal 2020)
RNase P Probe	TTCTGACCTGAAGGCTCTGCGCG	(Lu et al. Emerging Infectious Disease journal 2020)
Nsp14 Forward	TGGGGYTTTACRGGTAACCT	(Chu et al. Clinical Chemistry 2020)
Nsp14 Reverse	AACRCGCTTAACAAAGCACTC	(Chu et al. Clinical Chemistry 2020)
Hypoxanthine phosphoribosyl transferase (HPRT) Forward	CAACAGGCTTTTCCTGGTT	(Ahmed et al. Cancer Genomics Proteomics 2007)
Hypoxanthine phosphoribosyl transferase (HPRT) Reverse	GGCTACTCTGCCCATGAAGA	(Ahmed et al. Cancer Genomics Proteomics 2007)
ß-actin Forward	CCCAGCACAATGAAGATCAA	(Yu et al. Cancer Epidemiology Biomarkers & Prevention 2008)
ß-actin Reverse	ACATCTGCTGGAAGGTGGAC	(Yu et al. Cancer Epidemiology Biomarkers & Prevention 2008)
Glyceraldehyde 3-phosphate dehydrogenase (GAPDH) Forward	GTCGTGGAGTCTACTGGTGTCTTC	(Tricarico et al. Anal Biochem 2002)
Glyceraldehyde 3-phosphate dehydrogenase (GAPDH) Reverse	GTCATATTTCTCGTGGTTCACACC	(Tricarico et al. Anal Biochem 2002)
16S 8F (qPCR control)	AGAGTTTGATCCTGGCTCAG	(Frank et al. Appl Environ Microbiol 2008)
16S 1391R (qPCR control)	GACGGGCGGTGWGTRCA	(Frank et al. Appl Environ Microbiol 2008)

Supplementary Table 5 (Spreadsheet)
This table provides complete de-identified clinical metadata for each COVID-19 patient included in this study.

Supplementary Table 6 (Spreadsheet)
This table provides de-identified raw source data from each of the assays presented in this manuscript.

Supplementary Table 7 (Spreadsheet)
This table provides information to aid interpretation of Supplementary Table 6.

Supplemental Item 1

Clinical Questionnaire:

Simple questionnaire:

1. Diarrhea (Y/N)?
2. Nausea (Y/N)?
3. Vomiting (Y/N)?

Detailed questionnaire:

1. Are you having diarrhea? (Y/N)
2. When did your diarrhea start? (date)
3. When did any of your other symptoms start including fever or other symptoms (cough, shortness of breath, sore throat? (date)
4. Did your diarrhea end or is it ongoing?
5. How many stools are you having per day?
6. Is there any blood in your stool? (Y/N)
7. Are stools large volume and mostly water? (Y/N)
8. Are you waking up in the middle of the night to have bowel movement? (Y/N)
9. Do you have abdominal pain? (Y/N)

Other GI symptoms / history question:

1. Is your appetite decreased? (Y/N)
2. Are you having nausea? (Y/N)
3. Are you having any episodes of vomiting? (Y/N)

Supplementary References

Ahmed, F. E., Vos, P., iJames, S., Lysle, D. T., Allison, R. R., Flake, G., Sinar, D. R., Naziri, W., Marcuard, S. P. and Pennington, R. (2007). Transcriptomic molecular markers for screening human colon cancer in stool and tissue. Cancer Genomics Proteomics **4**(1): 1-20.

Chu, D. K. W., Pan, Y., Cheng, S. M. S., Hui, K. P. Y., Krishnan, P., Liu, Y., Ng, D. Y. M., Wan, C. K. C., Yang, P., Wang, Q., et al. (2020). Molecular Diagnosis of a Novel Coronavirus (2019-nCoV) Causing an Outbreak of Pneumonia. Clinical Chemistry **66**(4): 549-555.

Frank, J. A., Reich, C. I., Sharma, S., Weisbaum, J. S., Wilson, B. A. and Olsen, G. J. (2008). Critical evaluation of two primers commonly used for amplification of bacterial 16S rRNA genes. Appl Environ Microbiol **74**(8): 2461-2470.

Lu, X., Wang, L., Sakthivel, S., Whitaker, B., Murray, J., Kamili, S., Lynch, B., Malapati, L., Burke, S., Harcourt, J., et al. (2020). US CDC Real-Time Reverse Transcription PCR Panel for Detection of Severe Acute Respiratory Syndrome Coronavirus 2. Emerging Infectious Disease journal **26**(8): 1654.

Tricarico, C., Pinzani, P., Bianchi, S., Paglierani, M., Distante, V., Pazzagli, M., Bustin, S. A. and Orlando, C. (2002). Quantitative real-time reverse transcription polymerase chain reaction: normalization to rRNA or single housekeeping genes is inappropriate for human tissue biopsies. Anal Biochem **309**(2): 293-300.

Yu, Y. J., Majumdar, A. P. N., Nechvatal, J. M., Ram, J. L., Basson, M. D., Heilbrun, L. K. and Kato, I. (2008). Exfoliated Cells in Stool: A Source for Reverse Transcription-PCR–Based Analysis of Biomarkers of Gastrointestinal Cancer. Cancer Epidemiology Biomarkers & Prevention **17**(2): 455.
